# Supplementary material for: Happy just because. A cross-cultural study on subjective wellbeing in three Indigenous societies
Source: PLoS One. 2021 May 13;16(5):e0251551. doi: 10.1371/journal.pone.0251551 (PMC8118246; doi:10.1371/journal.pone.0251551)
Supplement: S1 Protocol — (DOCX) [file pone.0251551.s001.docx]

**Supporting Information**

**Protocol for the questions on “wellbeing”**

Timing: Administer **once each quarter**

Sample: Data on wellbeing should be collected from all adults (18 years and over) willing to participate.

Protocol:

- As much as possible, ask the question in an open-ended way (i.e., in a free-flowing conversation), rather than as a close-ended survey.
- Allow for open-ended answers, as respondents may want to explain in some words or sentences how they are feeling, and make the judgment yourself as to which category their answer fits best. Really listen to what respondents have to say; be respectful and considerate.
- Some answers might require further probing or explaining (e.g., giving examples in which context people might have felt different feelings, or emphasising when a question is mean to ask them about their state “in general”). This requires certain skill. Be as objective and “vague” as possible, not putting more emphasis on one possible answer than on another, so as not to direct the person to answer only in reference to one aspect of their life.
- Remember that you should write down the exact answer they give to you, keeping track of a free list of answers as you go along, and assign codes later.
- Also remember that possible answers include on “do not want to report” (-11) and “do not know” (-9).

**Tbl_wellbeing**

| **Code** | **Instructions** | **Format** | **0000000** | **0000000** |
| --- | --- | --- | --- | --- |
| idsubjid | Personal identification number | 9 digits |  |  |
| *imind* | Taking everything into consideration, would you say your life is?  Translation into Baka: *A sia e kope, mu a doto pe?*  Translation into Bahasa Indonesia: *Secara umum, apakah kamu.... (bahagia).*  Translation into Tsimane’: *Juñi buty tyi mi?* | *0=very bad;*  *1=not good; 2=fair;*  *3=good;*  *4=very good* |  |  |
| *Imindcause* | Why so? (reason/explanation to previous answer) | *text* |  |  |
